# Supplementary material for: The association between alcohol intake and incident atrial fibrillation in older adults: The ARIC cohort
Source: PLoS One. 2024 Nov 21;19(11):e0314207. doi: 10.1371/journal.pone.0314207 (PMC11581337; doi:10.1371/journal.pone.0314207)
Supplement: S1 Table — aAdjusted for age, sex, race, education level, prevalent cardiovascular disease [coronary artery disease (CAD), heart failure (HF), and stroke], hypertension (HTN), HDL-C, LDL-C, use of antihypertensive medications, use of anticoagulants, diabetes, smoking status, and body mass index (BMI). (DOCX) [file pone.0314207.s001.docx]

**Supplemental Table S1.** Risk of incident atrial fibrillation by quartiles of years of abstinence in former drinkers (n=1,393)

|  | **Unadjusted Hazard Ratio** | **95% Confidence Interval** | **Adjusted Hazard Ratio^a^** | **95% Confidence Interval** |
| --- | --- | --- | --- | --- |
| **Quartile 1 (0-8 yrs)** | 1 (Ref.) | Ref. | 1 (Ref.) | Ref. |
| **Quartile 2 (9-25 yrs)** | 1.30 | 0.92-1.84 | 1.33 | 0.93-1.90 |
| **Quartile 3 (26-38 yrs)** | 1.07 | 0.68-1.67 | 0.77 | 0.50-1.17 |
| **Quartile 4 (39-75 yrs)** | 1.00 | 0.68-1.48 | 0.67 | 0.47-0.96 |

^a^ Adjusted for age, sex, race, education level, prevalent cardiovascular disease [coronary artery disease (CAD), heart failure (HF), and stroke], hypertension (HTN), HDL-C, LDL-C, use of antihypertensive medications, use of anticoagulants, diabetes, smoking status, and body mass index (BMI).
