# Supplementary material for: The association between alcohol intake and incident atrial fibrillation in older adults: The ARIC cohort
Source: PLoS One. 2024 Nov 21;19(11):e0314207. doi: 10.1371/journal.pone.0314207 (PMC11581337; doi:10.1371/journal.pone.0314207)
Supplement: S2 Table — aAdjusted for age, sex, race, education level, prevalent cardiovascular disease [coronary artery disease (CAD), heart failure (HF), and stroke], hypertension (HTN), HDL-C, LDL-C, use of antihypertensive medications, use of anticoagulants, diabetes, smoking status, and body mass index (BMI). (DOCX) [file pone.0314207.s002.docx]

**Supplemental Table S2.** Risk of incident atrial fibrillation by 20-year intervals of years in former drinkers (n=1,393)

|  | **Unadjusted Hazard Ratio** | **95% Confidence Interval** | **Adjusted Hazard Ratio*** | **95% Confidence Interval** |
| --- | --- | --- | --- | --- |
| **0-20 yrs** | 1 (Ref.) | Ref. | 1 (Ref.) | Ref. |
| **21-40 yrs** | 0.91 | 0.66-1.24 | 0.84 | 0.61-1.15 |
| **41-60 yrs** | 0.92 | 0.59-1.42 | 0.87 | 0.56-1.35 |
| **61-80 yrs** | 1.26 | 0.47-3.46 | 0.80 | 0.28-2.27 |

^a^ Adjusted for age, sex, race, education level, prevalent cardiovascular disease [coronary artery disease (CAD), heart failure (HF), and stroke], hypertension (HTN), HDL-C, LDL-C, use of antihypertensive medications, use of anticoagulants, diabetes, smoking status, and body mass index (BMI).
