# Supplementary material for: The association between alcohol intake and incident atrial fibrillation in older adults: The ARIC cohort
Source: PLoS One. 2024 Nov 21;19(11):e0314207. doi: 10.1371/journal.pone.0314207 (PMC11581337; doi:10.1371/journal.pone.0314207)
Supplement: S3 Table — aAdjusted for age, sex, race, education level, prevalent cardiovascular disease [coronary artery disease (CAD), heart failure (HF), and stroke], hypertension (HTN), HDL-C, LDL-C, use of antihypertensive medications, use of anticoagulants, diabetes, smoking status, and body mass index (BMI). (DOCX) [file pone.0314207.s003.docx]

**Supplemental Table S3.** Risk of incident atrial fibrillation by quartiles of years of drinking in former drinkers (n=676)

|  | **Unadjusted Hazard Ratio** | **95% Confidence Interval** | **Adjusted Hazard Ratio*** | **95% Confidence Interval** |
| --- | --- | --- | --- | --- |
| **Quartile 1 (0-5 yrs)** | 1 (Ref.) | Ref. | 1 (Ref.) | Ref. |
| **Quartile 2 (6-10 yrs)** | 1.59 | 0.78-3.27 | 1.67 | 0.80-3.48 |
| **Quartile 3 (11-20 yrs)** | 1.47 | 0.75-2.91 | 1.59 | 0.79-3.17 |
| **Quartile 4 (21-43 yrs)** | 1.21 | 0.6-2.36 | 1.19 | 0.60-2.35 |

^a^ Adjusted for age, sex, race, education level, prevalent cardiovascular disease [coronary artery disease (CAD), heart failure (HF), and stroke], hypertension (HTN), HDL-C, LDL-C, use of antihypertensive medications, use of anticoagulants, diabetes, smoking status, and body mass index (BMI).
