# Supplementary material for: The association between alcohol intake and incident atrial fibrillation in older adults: The ARIC cohort
Source: PLoS One. 2024 Nov 21;19(11):e0314207. doi: 10.1371/journal.pone.0314207 (PMC11581337; doi:10.1371/journal.pone.0314207)
Supplement: S4 Table — aAdjusted for age, sex, race, education level, prevalent cardiovascular disease [coronary artery disease (CAD), heart failure (HF), and stroke], hypertension (HTN), HDL-C, LDL-C, use of antihypertensive medications, use of anticoagulants, diabetes, smoking status, and body mass index (BMI). bThe category of 31–43 years includes 1 participant who had >40 years of drinking and was not separated into an additional category due to low sample size. All other participants had between 31–40 years of drinking. (DOCX) [file pone.0314207.s004.docx]

**Supplemental Table S4.** Risk of incident atrial fibrillation by 10-year intervals for years of drinking in former drinkers (n=676)

|  | **Unadjusted Hazard Ratio** | **95% Confidence Interval** | **Adjusted Hazard Ratio^a^** | **95% Confidence Interval** |
| --- | --- | --- | --- | --- |
| **0-10 yrs** | 1 (Ref.) | Ref. | 1 (Ref.) | Ref. |
| **11-20 yrs** | 0.94 | 0.56-1.58 | 0.88 | 0.52-1.51 |
| **21-30 yrs** | 1.49 | 0.85-2.60 | 1.19 | 0.65-2.16 |
| **31-43 yrs^b^** | 1.93 | 0.90-4.14 | 1.86 | 0.81-4.26 |

^a^ Adjusted for age, sex, race, education level, prevalent cardiovascular disease [coronary artery disease (CAD), heart failure (HF), and stroke], hypertension (HTN), HDL-C, LDL-C, use of antihypertensive medications, use of anticoagulants, diabetes, smoking status, and body mass index (BMI).

^b^ The category of 31-43 years includes 1 participant who had >40 years of drinking and was not separated into an additional category due to low sample size. All other participants had between 31-40 years of drinking.
