# Supplementary material for: The association between alcohol intake and incident atrial fibrillation in older adults: The ARIC cohort
Source: PLoS One. 2024 Nov 21;19(11):e0314207. doi: 10.1371/journal.pone.0314207 (PMC11581337; doi:10.1371/journal.pone.0314207)
Supplement: Attachment — Submitted filename: Response to Reviewers_2.docx [file pone.0314207.s005.docx]

To whom it may concern:

Please see below for responses to journal requirements and reviewer comments.

**Journal Requirements**

1. PLOS ONE style requirements

Title and manuscript body changed to meet style requirements.

2. Funding Statement

Included additional clarifications on all author funding. Below is the updated funding statement: The Atherosclerosis Risk in Communities study has been funded in whole or in part with Federal funds from the National Heart, Lung, and Blood Institute, National Institutes of Health, Department of Health and Human Services, under Contract nos. (75N92022D00001, 75N92022D00002, 75N92022D00003, 75N92022D00004, 75N92022D00005). There was no additional funding received for this study. The authors declare no competing interests.

3. Data availability

All data is freely available to other researchers in a public repository. Below is the updated data availability statement which was used in a previously published ARIC study in PLOS ONE: Peter-Marske et al. (2024). PMID: 39083538.

Data can be requested through the Biologic Specimen and Data Repository Information Coordinating Center (BioLINCC) website ([https://biolincc.nhlbi.nih.gov/studies/aric/](https://nam11.safelinks.protection.outlook.com/?url=https%3A%2F%2Fbiolincc.nhlbi.nih.gov%2Fstudies%2Faric%2F&data=05%7C02%7Clouis.li%40emory.edu%7C657e491b53954b12008d08dcc900ea50%7Ce004fb9cb0a4424fbcd0322606d5df38%7C0%7C0%7C638606252964765751%7CUnknown%7CTWFpbGZsb3d8eyJWIjoiMC4wLjAwMDAiLCJQIjoiV2luMzIiLCJBTiI6Ik1haWwiLCJXVCI6Mn0%3D%7C0%7C%7C%7C&sdata=6Q5hN2LTC2xxet460kJ%2BCkFN1M87l2xefWDKg0%2BINtk%3D&reserved=0)) after creating an account and registering with the site. The data dictionary is available on this website. More information about the ARIC study can be found at [https://aric.cscc.unc.edu/aric9/](https://nam11.safelinks.protection.outlook.com/?url=https%3A%2F%2Faric.cscc.unc.edu%2Faric9%2F&data=05%7C02%7Clouis.li%40emory.edu%7C657e491b53954b12008d08dcc900ea50%7Ce004fb9cb0a4424fbcd0322606d5df38%7C0%7C0%7C638606252964778585%7CUnknown%7CTWFpbGZsb3d8eyJWIjoiMC4wLjAwMDAiLCJQIjoiV2luMzIiLCJBTiI6Ik1haWwiLCJXVCI6Mn0%3D%7C0%7C%7C%7C&sdata=AGgXLs3Rkifv3%2BJgq8oVL3%2BYiL%2BT86Tp5bIDGRuLKRM%3D&reserved=0).

4. Data availability clarification

Are these third party data (i.e., data not owned or collected by the author(s))? If these are indeed third party data, please confirm that others would be able to access these data in the same manner as the authors and that the authors did not have any special access privileges that others would not have.

Yes, we confirm that these are third party data, and anyone would be able to access the data in the same manner as the authors. The authors did not have special access privileges that others would not have.

**Reviewer #1**
1. The study has limitations in determination of the alcohol intake status (self-reported) and quantity of alcohol consumed (potential recall bias). This was appropriately acknowledged in the limitations.

Acknowledged and no response required.

2. There may also be problems in AF determination. The methodology of determining AF by ICD codes may miss paroxysmal episodes. I would be more explicit in acknowledging this limitation in the manuscript

Included language to explicitly acknowledge limitations of AF acquisition: *Next, due to incident AF being ascertained from ECGs and ICD codes and without echocardiographic data, the severity and type of AF, such as paroxysmal, persistent, or permanent were not captured.* ***In cases such as paroxysmal episodes, incident AF may be missed entirely.***

3. In the Discussion section authors have tried to explain alcohol’s negative association with AF among older individuals vs. positive association among younger individuals. One possibility could be that in the older individuals other, more powerful predictors of AF drawn weaker predictors such as alcohol.

Included language to clarify this statement: *In this study of older adults, other risk factors may be more powerful predictors of AF that led to weaker associations between alcohol intake and AF.*

4. Why does Figure 2 have 4 panels when panel A is providing the whole picture? Furthermore, changing the color scheme in each panel is confusing. I would only present panel A

The panels were initially provided to show the statistical significance of results between comparisons. Panel A showed that there was statistically significant difference when all 3 groups were compared together whereas Panels B-D show that the difference between the comparisons: current vs. former, current vs. never, and former vs. never. These additional panels show that there was not statistically significant difference between current vs. former and current vs. never drinkers, and that the main contributing reason for the statistically significant difference in Panel A is a result of the differences between former vs. never drinkers (Panel D, p<0.05).

Changed Figure 2 to present only Panel A with an updated caption and additional explanation in the text as follows: *With an alpha level of 0.05, there was a statistically significant difference in the risk of incident AF between categories due to the difference in risk between former and never drinkers (Fig 2). The difference in risk between former vs. never drinkers was statistically significant (p=0.006). The differences between current vs. former and current vs. never drinkers were not statistically significant (p=0.067 and 0.152, respectively).*

5. Line 356, “that” repeated twice

Removed “that”.

**Reviewer #2**

I have no particular concern about the manuscript. The argument is very interesting, the data are abundant and derive by a well-known study, English language is plain and clear, strength and limitations are well exposed.

Acknowledged and no response required.

**Reviewer #3**

1. No echocardiographic data to diagnose the AF etiology and presence of structural heart disease
Even though echocardiographic data was collected in a subset of ARIC participants at visit 5 (baseline for this analysis), we do not have echocardiographic data at the time of AF diagnosis. We understand this lack of data to characterize AF etiology and determine the presence of structural heart disease is a limitation and we now note it in the Discussion as a limitation: *Next, incident AF was ascertained from ICD codes and death certificates without ECG, Holter monitoring, or echocardiographic data. While this may affect our results by missing cases, such as in paroxysmal episodes where AF may be missed entirely, previous studies have validated the use of hospital discharge codes and death certificates for AF ascertainment.*

2. Thrombotic risk as CHADS VASC score and presentation with systemic embolization as CVA is not included.
The primary focus of our analysis is on the link between alcohol consumption and development of new onset AF, rather than on the risk of thromboembolic events in people with AF. Thus, inclusion of the CHA2DS2-Vasc score and consideration of thromboembolic outcomes is not directly relevant to our primary study question.

3. No documented evidence of presence of AF as ECG or holter monitoring.

Prevalent AF was ascertained via ECGs performed at study visits and hospital discharge codes prior to baseline for this analysis, as Holter monitoring was not conducted as part of the ARIC examinations. Similarly, incident AF relied on hospital discharge codes and death certificates. This is not the gold standard for AF diagnosis, which would require evidence of the arrhythmia in an ECG recording. However, validation studies, including our work in the ARIC cohort (Alonso et al, 2009, PMID: 19540400; Jensen et al, 2012, PMID: 22262600), have demonstrated the excellent validity of this approach to identify AF in large epidemiologic studies in which Holter monitoring and repeated ECGs is not logistically feasible. We mention lack of ECG confirmation of all AF cases and Holter monitoring in the study as limitations that could affect our results: *Next, incident AF was ascertained from ICD codes and death certificates without ECG, Holter monitoring, or echocardiographic data. While this may affect our results by missing cases, such as in paroxysmal episodes where AF may be missed entirely, previous studies have validated the use of hospital discharge codes and death certificates for AF ascertainment.^19,28^ Data on the severity and type of AF, such as paroxysmal, persistent, or permanent were not captured, and these characteristics may be important distinctions in the effects of alcohol consumption on AF incidence.*
